# Supplementary material for: Knowledge, Attitudes and Practices of Eye Health among Public Sector Eye Health Workers in South Africa
Source: Int J Environ Res Public Health. 2021 Nov 27;18(23):12513. doi: 10.3390/ijerph182312513 (PMC8656467; doi:10.3390/ijerph182312513)
Supplement: Supplementary file 1 [file ijerph-18-12513-s001.zip › ijerph-1474493-supplementary.pdf]

Variables	Number of items	mean	SD	Cronbach's alpha
Knowledge	21	3.11	0.50	0.72
Attitude	21	2.38	0.38	0.85
Perceptions	22	1.62	0.40	0.84

Reliability analysis outputs: I used Cronbach alpha, in order to check the reliability (i.e. consistency of the items). According to the outputs, the items of knowledge, attitude, and perceptions are consistent (i.e. reliable) because the corresponding alpha greater than 0.60.
